# Utility and Micro-Costing Framework for *TERT* Promoter Mutation Analysis in Melanocytic Lesions of Uncertain Malignant Potential: A Retrospective Study in Dutch Local Clinical Practice

**DOI:** 10.3390/diagnostics14151665

**Published:** 2024-08-01

**Authors:** Leire Barrutia, Ed Schuuring, Emõke Rácz, Gilles F. H. Diercks, Léon C. van Kempen

**Affiliations:** 1 Department of Pathology and Medical Biology, University Medical Center Groningen, 9713 GZ Groningen, The Netherlands; 2 Department of Dermatology, Medicine and Toxicology, University of Valladolid, 47002 Valladolid, Spain; 3 Department of Dermatology, University Medical Center Groningen, 9713 GZ Groningen, The Netherlands; 4 Department of Pathology, University of Antwerp, Antwerp University Hospital, 2650 Edegem, Belgium

**Keywords:** melanocytic tumors, *TERT*, micro-costing framework

## Abstract

The 2018 WHO edition on the classification of cutaneous melanocytic tumors recognizes eight evolutionary pathways of melanoma and describes tumors of uncertain malignant potential for each. When histology and immunohistochemistry do not support a confident conclusion about its malignant potential, a window of diagnostic uncertainty is created. Mutations in the telomerase reverse transcriptase gene promoter (*TERT*p) are highly specific for melanoma and can be used as an ancillary technique to acquire a higher level of confidence in the diagnosis. However, little is known about the cost-effectiveness of testing for *TERT*p mutations. The aims of this study were to determine how often knowledge of the *TERT*p mutation status contributed to the final diagnosis and to develop a micro-costing framework to calculate cost-effectiveness. A retrospective analysis of all cutaneous melanocytic lesions that were discussed in the Noord-Nederland Melanoma Panel from January 2021 to October 2022 was performed to identify the cases in which the preliminary histopathological diagnosis was uncertain regarding malignancy (ambiguous, likely benign, or likely malignant). For cases in which a *TERTp* mutation analysis was performed, the final diagnoses were collected, and it was determined whether this impacted the overall conclusion. A micro-costing framework was established to model the financial impact of introducing *TERTp* mutation analyses and subsequent clinical procedures. The study included 367 cases, of which 175 diagnoses of uncertain malignant potential were initially reported. *TERTp* mutation analysis was performed for 151/175 (86%). In 38% of these cases, a higher level of confidence regarding malignant potential was obtained. The implementation of *TERT*p mutation analyses for cutaneous melanocytic proliferations with uncertain malignant potential can narrow the window of diagnostic uncertainty. For the patient group with an initial uncertain diagnosis, the increased cost for molecular testing (86.145 €) was compensated by a reduced overall treatment cost (−122.304 €). A microsimulation model to determine the cost-effectiveness of *TERT*p mutation analysis projected an overall saving for the healthcare system.

## 1. Introduction

The 2018 WHO classification of melanocytic tumors describes distinct evolutionary pathways of melanoma, seven of which are for cutaneous melanoma [1,2,3]. The pathways are divided into low cumulative sun damage (low-CSD) melanoma, corresponding to superficial spreading melanoma, and high-CSD melanoma, which includes lentigo maligna melanoma, desmoplastic melanoma, Spitz melanoma, acral melanoma, melanoma in blue nevus, and melanoma in congenital nevus [1,2,3,4]. In each of these pathways, progressive multistep morphological and molecular evolution from a benign nevus to a melanoma is proposed, associated with the acquisition of increasing genomic abnormalities [1,2,3,4,5].

The intermediate steps in each pathway comprise melanocytic lesions of uncertain malignant potential. Some of these lesions can present with histopathological signs of atypia but overall seem likely benign, such as dysplastic nevi or atypical spitz tumors. Other lesions present with numerous signs of atypia and are perceived as likely malignant but fall short of a definitive diagnosis of melanoma. A third group of these lesions stand in the middle and are perceived as ambiguous by pathologists. Consequently, the latter are subclassified into subjective, descriptive categories whose definitions are not linked to specific morphologic subsets of melanocytic proliferations [4]. These categories include ‘intraepidermal atypical melanocytic proliferation of uncertain significance’ (IAMPUS), which poses a differential diagnosis with melanoma in situ, ‘superficial atypical melanocytic proliferation of uncertain significance’ (SAMPUS), which must be distinguished from early invasive, radial growth phase melanoma, and ‘melanocytic tumor of uncertain malignant potential’ (MELTUMP), which requires differentiation from melanoma in the vertical growth phase [1,2,3,4]. 

These uncertain diagnoses have important clinical implications. Robust evidence is lacking regarding their management, including the optimal excision margins, the need for sentinel node biopsy, and the need for follow-up [6]. Due to the potential lethality of melanoma, a cautious attitude is generally adopted in these cases [7]. This could potentially lead to unnecessary surgical procedures for margin re-excision and sentinel node biopsy, unnecessary imaging tests to exclude dissemination, and unnecessary follow-up that can result in a substantial and avoidable increase in health system costs. Therefore, the previous literature has highlighted the importance of performing additional immunohistochemical and molecular diagnostic techniques to properly determine the malignant potential of these lesions and efficiently establish the optimal clinical approach [4]. 

Molecular diagnostics constitute an objective and reproducible diagnostic tool. In particular, although their prevalence is variable in different studies, telomerase reverse transcriptase promoter (*TERT*p) mutations have been found to be prevalent in melanoma and are very rare in benign melanocytic lesions [8,9,10]. In melanoma, the most common mutations in the *TERT*p are c.−124 C > T (also known as C228T) and c.−146 C > T (also known as C250T), which are associated with poor clinical outcomes, worse prognosis, and reduced survival [8,9,10,11,12,13,14,15]. In addition, the less frequent c.−138_−139delinsTT mutation is associated with the worst survival in stage I and II melanoma [16].

Due to the positive predictive value of *TERT*p C228T and C250T for malignancy (98.5%), *TERT*p mutation analysis can be used as ancillary evidence to classify uncertain melanocytic lesions [2,4,14].

Nonetheless, this technique has two relevant limitations. First, it has a reported sensitivity of 77.9% and a low negative predictive value, which means that a negative result cannot exclude malignancy [14]. Second, mutation analysis via droplet digital PCR or NGS can be cost-prohibitive. To date, there is limited evidence to support that the increased costs of molecular testing outweigh the total costs associated with the clinical implications of melanocytic lesions with a diagnosis of uncertain malignant potential. 

The current study was designed to assess the value of *TERT*p mutation analysis in reducing diagnostic uncertainty based on the experience of a Dutch melanoma panel in a clinical setting, and to develop a micro-costing framework to objectively evaluate the benefit of performing this molecular diagnostic technique from a health system cost perspective. 

## 2. Materials and Methods

### 2.1. Analysis of the Effectiveness of TERTp Mutation Analysis in Reaching a Definitive Diagnosis for Melanocytic Lesions of Uncertain Malignant Potential

#### 2.1.1. Data Collection

All the cases discussed in the Noord-Holland Melanoma Panel from January 2021 to October 2022 were included (367 cases). This panel convenes monthly in a reference center in a tertiary university hospital and receives consultations about challenging melanocytic lesions from four hospitals in the north of the Netherlands. The panel is comprised of 8 dermatopathologists from the consulting centers and 2 dermatopathologists from the reference center. 

A retrospective analysis of the pathology reports was performed to identify the cases in which preliminary histopathological diagnosis was uncertain regarding malignancy. The preliminary histopathological diagnosis had to be agreed upon among all the dermatopathologists on the melanoma panel. The cases were classified into ambiguous, likely benign, or likely malignant. A case was classified as likely benign when the preliminary histopathological diagnosis was dysplastic nevus, acral nevus with dysplasia, or preferentially Spitz nevus versus an atypical spitzoid tumor. A case was classified as ambiguous when the preliminary histopathological diagnosis was IAMPUS (intraepidermal atypical melanocytic proliferation of uncertain significance), SAMPUS (superficial atypical melanocytic proliferation of uncertain significance), or MELTUMP (melanocytic tumor of uncertain malignant potential). A case was classified as likely malignant when the preliminary histopathological diagnosis was preferentially a melanoma versus an intermediate lesion of uncertain malignant potential. The histopathological data, immunohistochemical results, and *TERT*p mutation status were retrieved from the pathology reports, along with the panel’s final conclusion regarding the interpretation of these combined results and the final recommendation. 

#### 2.1.2. Descriptive Analysis of the Case Series

Demographic analyses of the case series were performed to describe basic features of the cases, including sex, age, and location of the melanocytic lesions. 

#### 2.1.3. *TERT*p Mutation Analysis

Four formalin-fixed paraffin-embedded tissue sections (10 µm) were cut and mounted on superfrost slides (ThermoFisher Scientific, Waltham, MA, USA). The slides were dewaxed with Xylene and rehydrated through graded ethanol steps. Guided by an adjacent 4 µm section that was haematoxilin–eosin-stained, manual macrodisssection of the region of interest containing at least 5% neoplastic cells was performed. The sample was transferred to a microcentrifuge tube. DNA extraction was performed using a Maxwell RSC DNA FFPE kit and a Maxwell RSC system (Promega, Leiden, The Netherlands), according to the manufacturer’s instructions, and was eluted in 60 µL TE-4. 

Droplet digital PCR (ddPCR) analysis using the Bio-Rad QX200 system was performed to determine the presence of *TERT* (NM_198253.3) c.−124 C > T (C228T) and c.−146 C > T (C250T) mutations. Assays were purchased from Bio-Rad: TERT C228T ddPCR™ Expert Design Assay: TERT C228T_113 (Assay ID dHsaEXD72405942; Tm: 62 C; ramp rate 2.5 C/s; 2D amplitude horizontal and vertical threshold values: 1500 and 3000; positive control cell line: HepG2; negative control cell line: A549) and TERT C250T ddPCR™ Expert Design Assay: TERT C250T_113 (Assay ID dHsaEXD46675715; Tm: 62 C; ramp rate 2.5 C/s; 2D amplitude horizontal and vertical threshold values: 2000/1500; positive control cell line: SiHa; negative control cell line: A549). PCR and droplet generation was performed according to the manufacturer’s instructions. The assay was validated to call a TERT C228T or C250T mutation if ≥6 mutant droplets were detected and negative if <6 mutant droplets with at least 600 total positive (wild-type and mutant) droplets were detected (ensuring an analytical sensitivity of at least 1%). 

All the procedures were performed under ISO-15189 accreditation [17] All standard precautions were taken to avoid contamination of the amplification products using separate laboratories for pre- and post-PCR handling.

#### 2.1.4. Analysis of the Modifying Effect of *TERT*p Mutation Status on the Preliminary Diagnosis

The modifying effect of the *TERT*p mutation analysis on the a priori histopathological diagnosis was studied. To estimate the predictive probability that a lesion had malignant potential, the a priori histopathological diagnosis was combined with *TERT*p mutation status using a Bayesian approach [18].

In order to achieve this, we employed a formula based on the Bayes rules to calculate the conditional probability that the lesion was malignant when a *TERT*p mutation was detected, described as P (malignant|*TERT*p mutation status+), and the conditional probability that lesion was malignant when a *TERT*p mutation was not detected, described as P (malignant|*TERT*p mutation status−), giving rise to the following two equations:

(1) $$P\left(malignant|TERTp\;mutation\;status\;+\right)=\;\frac{P\left(TERTp\;mutation\;status\;+\right|malignant)\times P(malignant)}{P\left(TERTp\;mutation\;status\;+\right|malignant)\times P\left(malignant\right)+P\left(TERTp\;mutation\;status\;+\right|benign)\times (1-P(malignant)}$$

(2) $$P\left(malignant|TERTp\;mutation\;status\;-\right)=\;\frac{P\left(TERTp\;mutation\;status\;-\right|malignant)\times P(malignant)}{P\left(TERTp\;mutation\;status\;-\right|malignant)\times P\left(malignant\right)+P\left(TERTp\;mutation\;status\;-\right|benign)\times (1-P(malignant)}$$

The probability P (malignant|*TERT*p mutation status+) can be calculated when a value for the following arguments can be estimated: (1) P (*TERT*p mutation status+|Malignant), the probability of a positive *TERT*p mutation status given that the lesion is malignant; (2) P (*TERT*p mutation status+|Benign), the probability of a positive *TERT*p mutation status given that the lesion is benign; and (3) P (malignant), the a priori probability that the lesion is malignant vs. benign. The same applies to the second equation, regarding the probability P (malignant|*TERT*p mutation status−). 

To determine the probabilities P (*TERT*p mutation status+|Malignant) and P (*TERT*p mutation status−|Malignant), a literature review was conducted. The results of the prevalence of *TERT*p mutations in melanomas varied widely across different studies. Gandini et al. [9] performed a systematic review and meta-analysis in 2021. This data was used to calculate the probabilities P (*TERT*p mutation status+|Malignant) and P (*TERT*p mutation status−|Malignant) (Table 1). 

Similarly, a literature review was conducted to determine the probabilities P (*TERT*p mutation status+|Benign) and P (*TERT*p mutation status−|Benign). Thomas et al. [12] analyzed the largest series of melanocytic nevi for *TERT*p mutations in 2018 and found that 1.4% of melanocytic nevi harbored *TERT*p mutations. Therefore, we estimated the probability P (*TERT*p mutation status+|Benign) as 0.014, and the probability P (*TERT*p mutation status−|Benign) as 0.986. 

The a priori histopathological diagnosis was considered to calculate the probability that a lesion was malignant P (malignant). When a lesion was classified as likely benign, P (malignant) was set at 0.25. When classified as ambiguous or likely malignant, P (malignant) was set at 0.5 or 0.75, respectively. 

### 2.2. Micro-Costing Framework 

#### 2.2.1. Design

The goal was to determine the utility of performing the *TERT*p mutation analysis from a health system cost perspective. For this purpose, a micro-costing framework was developed to assess the impact of *TERT*p mutation analysis. The framework compared two scenarios. In the first scenario, the total costs derived from lesions being classified as uncertain before performing *TERT*p mutation analysis were calculated. In the second scenario, the cost of performing *TERT*p mutation analysis and the costs derived from the remaining uncertain lesions were added. The costs derived from malignant lesions, including re-excision, staging, follow-up, and therapeutic costs, were not taken into account in this framework as they were considered to be indicated and unavoidable.

The foundation of the framework was based on the two Implicated processes: (i) Ie step-wise diagnostic workflow of the *TERT*p mutation analysis and I the actions undertaken in clinical management in response to the diagnosis of a melanocytic lesion of uncertain malignant potential. To calculate the cost of the first process, the activities required in the step-wise diagnostic workflow were determined. These activities included sample collection, transportation, processing, and the *TERT*p mutation analysis. To calculate the cost of the second process, the clinical management actions required were determined. These actions were based on the Melanoma Multidisciplinary Care Trajectory guidelines from the Noord-Oost Nederland region, which was developed in accordance with: ^(i)^ the 3rd version of the National Guidelines of Melanoma of the Netherlands [29], ^(ii)^ the 11th version of the Stichting Oncologische Samenwerking (SONCOS, Oncological Collaboration Foundation) standardisation report [30], and ^(iii)^ the 8th edition of the American Joint Committee on Cancer (AJCC) guidelines [31]. 

In the case of the *TERT*p mutation analysis, for each of the activities, the costs for personnel, materials, equipment, overheads, and failures were considered. The personnel costs were based on the estimated minutes spent per activity and the wage of the staff members performing the activity. The material costs were based on the materials consumed per activity. The equipment costs were based on the depreciation and maintenance costs of the equipment used per activity [32]. All costs were included in the per sample cost for the ddPCR analysis of the two *TERT*p mutations (i.e., c.124 C > T and c.−145 C > T). 

In the case of melanocytic lesions remaining uncertain, the Melanoma Multidisciplinary Care Trajectory guidelines from the Noord-Oost Nederland region established that the action required consisted of 5 mm margin re-excision surgery. The cost of this surgery was based on the tariff established by the hospital for dermatological surgery. The guidelines and the latest literature [2,6] establish that no further diagnostic techniques or follow-up are required in these cases.

#### 2.2.2. Analysis

**Figure 1:**
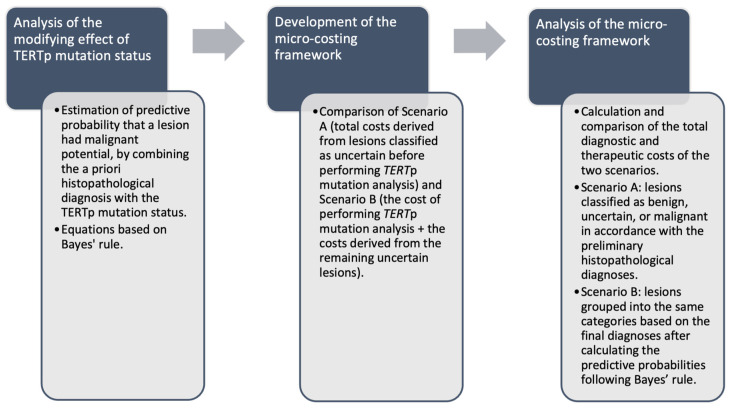
Methodology.

After designing the processes (Figure 1) and determining the costs for each of them, the total diagnostic and therapeutical costs were calculated and compared for the two scenarios. In the first scenario (A), the lesions were classified as benign, uncertain, or malignant in accordance with the preliminary histopathological diagnoses. In the second scenario (B), they were grouped into the same categories based on the final diagnoses after performing the *TERT*p mutation analysis and the predictive probabilities were calculated following Bayes’ rule. 

## 3. Results

### 3.1. Demographics

The 367 cases included in this study corresponded to 225 women and 142 men. The mean age was 44.3 years and the median age was 43 years. Most lesions were located on the extremities (41.7%), followed by the trunk (32.7%), the head and neck (14.2%), and acral areas (11.2%) (Table 2). 

#### 3.1.1. Lesion Classification According to Preliminary Histopathological Diagnosis

After conventional histopathological and immunohistochemical assessment of the 367 cases, 115 were considered benign, 77 were considered malignant, and 175 were considered uncertain. Within this last group, 87 were classified as ambiguous, 49 as likely benign, and 39 as likely malignant (Table 3). 

#### 3.1.2. Modifying Effect of the *TERT*p Mutation Analysis on the Preliminary Diagnosis 

The *TERT*p mutation analysis could be performed in 151 (86.3%) of the uncertain cases. Specifically, in 44 likely benign, 38 likely malignant, and 69 ambiguous lesions. In the remaining 24 uncertain cases (13.7%), the ddPCR was inconclusive due to analytical limitations (e.g., an insufficient sample). 

The *TERT*p mutation analysis (Table 4) did not identify a C228T or C250T mutation in 44/44 (100%) of the likely benign lesions. In the ambiguous group, a *TERT*p mutation was detected in 14/69 (20.3%) of the lesions. In the likely malignant group, a *TERT*p mutation was detected in 25/38 (65.8%) of the lesions (Table 4). In this series of cases, the C250T mutation was more prevalent than the C228T mutation (26/39 and 13/39, respectively, *p* < 0.01).

Following Bayes’ theorem to determine the probability of a diagnosis, the predictive probabilities of malignancy of a tumor with an a priori histopathological diagnosis and knowledge of the *TERT*p mutation status was calculated using Equations (1) and (2) (Table 5). The entries in the table demonstrate that when the *TERT*p mutation status was positive, malignancy was vastly more likely than benignity, regardless of the preliminary histopathological diagnosis. However, when the *TERT*p mutation status was negative, the a priori histopathological diagnosis was of high value. In this case, if the preliminary histopathological diagnosis was likely benign or ambiguous, benignity was more likely than malignancy. However, if the preliminary histopathological diagnosis was likely malignant, malignancy was more likely than benignity despite the absence of a *TERT*p mutation (Table 6). It was established that (1) when the probability of malignancy was above 0.5, the lesion was classified as likely malignant, (2) when the probability of malignancy was over 0.9, the lesion was classified as malignant, (3) when the probability of malignancy was under 0.2, the lesion was classified as likely benign, (4) when the probability of malignancy was under 0.1, the lesion was classified as benign, and (5) when the probability of malignancy ranged from 0.2 to 0.5, the lesion remained ambiguous, with the need for 5 mm re-excision. 

Table 5 displays the results of the calculations performed using Equations (1) and (2) to obtain the predictive probabilities of malignancy, based on the a priori histopathological diagnosis (likely benign = P (malignant) 0.25; ambiguous = P (malignant) 0.5; likely malignant = P (malignant) 0.75) and *TERT*p mutation status. 

The impact of the knowledge of the *TERT*p mutation status on the final diagnosis is visualized in Table 7. Of the 151 lesions grouped as uncertain after histopathological diagnosis (likely benign, ambiguous, and likely malignant), *TERT*p mutation analysis reclassified the lesions as benign (*n* = 44), ambiguous (*n* = 55), or malignant (*n* = 52). 

#### 3.1.3. Micro-Costing Framework 

Scenario A comprised 115 benign lesions, 77 malignant lesions, and 151 uncertain lesions. The total cost of 5 mm margin re-excisions of 151 uncertain lesions was 192.374 €. Scenario B comprised 159 likely benign lesions (+44), 55 uncertain lesions (−96), and 129 likely malignant lesions (+52). The total cost derived from performing the *TERT*p mutation analysis was 86.14550 €. The total cost of 5 mm margin re-excisions of 55 uncertain lesions was 70.070 €. Therefore, the global cost of Scenario B was 156.21550 €, which was 36.15850 € cheaper than Scenario A (Table 8). The cost of performing re-excisions for malignant lesions was not considered, as it is an unavoidable cost derived from an increase in diagnostic accuracy, resulting in the improvement of patient prognosis. 

Consequently, performing the *TERT*p mutation analysis contributed to reducing uncertainty by 38% (58/151) and lowered the global cost in our cohort of 371 patients by 36.158 €. 

## 4. Discussion

In this study, the modifying effect of *TERT*p mutation analysis on the final diagnosis of uncertain melanocytic lesions was assessed in real clinical practice, and a micro-costing framework was developed to determine its cost-effectiveness. 

Molecular diagnostics can be an objective and reproducible diagnostic tool in melanocytic lesions [33,34]. Particularly, the frequency of *TERT*p mutations exceeds the frequency of any known noncoding mutations in melanoma [8]. Due to its high positive predictive value for melanoma, it has a been proposed as reliable ancillary evidence for malignancy in uncertain melanocytic proliferations [14,15]. Furthermore, the latest studies have pointed out the emerging role of these mutations as possible therapeutic targets in melanoma [13,35]. However, the extensive use of this technique is limited by its cost. As a result, to date, the literature regarding its utility in real clinical practice is scarce, and systematic knowledge of its diagnostic value is lacking. Additionally, an analysis of the cost it generates overall in the management of uncertain melanocytic lesions in real clinical practice had not been performed. Within this framework, the aim of this study was to determine whether *TERT*p mutation analysis could be useful to narrow the window of diagnostic uncertainty in real clinical practice, and whether this could have a positive impact on total health system costs.

With the available data in the literature on the prevalence of *TERT*p mutations in cutaneous melanocytic tumors, we used a Bayesian approach to calculate the probability of malignancy. We found that *TERT*p mutation analysis resulted in a more confident diagnosis of benignity or malignancy in 38% of the uncertain melanocytic lesions tested. Specifically, it led to a diagnosis of malignancy in the 52/151 cases, allowing for the early detection of malignancy, adequate staging, and optimal management of melanoma in these cases, which is essential for improving patient outcomes and survival [13,36]. Similarly, it led to a definitive diagnosis of benignity in 44/151 cases, avoiding further unnecessary actions and thereby contributing to a reduction in health system costs. 

These results are line with the previous literature and highlight the high specificity and positive predictive value of this test for melanoma [14,15]. Following a Bayesian approach, we observed that positive *TERT*p status yields a very high probability that a melanocytic lesion is malignant, regardless of the preliminary histopathological diagnosis. Therefore, as hinted in the previous literature, it seems that performing this test is essential to improve diagnostic accuracy and the early detection of melanoma [13,14,15,35]. Additionally, our results show that, in the case of negative *TERT*p mutation status, the preliminary histopathological diagnosis is key in determining the probability of malignancy. In this case, although a lesion that is preliminarily classified as malignant will have a high probability of being malignant, a lesion that is preliminarily classified as benign or ambiguous will have a higher probability of being benign. In the case of an a priori ambiguous lesion, negative *TERT*p mutation status yields a probability of malignancy of 37%. This probability is more inclined towards benignity than towards malignancy, but it is not sufficient to rule out malignancy. Therefore, these lesions were still classified as ambiguous after performing the *TERT*p mutation analysis. In these circumstances, to narrow down uncertainty, clinical factors (e.g., phototype, dysplastic nevus syndrome, and familial melanoma) and additional histopathological prognostic factors, such as the proliferation index Ki67, should be considered. Vollmer [18] used Ki67 positivity in the context of Bayes’ theorem to determine the probability that a spitzoid lesion was a Spitz nevus or a melanoma.

Regarding the cost-effectiveness of the *TERT*p mutation analysis and its impact on healthcare economics, we designed two scenarios to compare the costs derived from the diagnoses of our case series before and after performing the *TERT*p mutation analysis. Implementing the *TERT*p mutation analysis resulted in a 19% reduction in the global cost associated with the management of our case series. We consider this finding of importance because it demonstrated that, despite low sensitivity, it is a cost-effective testing strategy with clinical and budgetary impacts. The latest literature regarding the clinical management of uncertain melanocytic lesions established that a margin re-excision of 5 to 10 mm must be performed in these cases [2,6]. In our setting, the cost of this surgery was 2.2 times higher than the cost of the test. Consequently, the decrease in the number of uncertain lesions classified as likely benign after performing the *TERT*p mutation analysis (44 lesions) resulted in a cost reduction of 36.158 € in our cohort of 151 patients with an initial ambiguous diagnosis.

Although specific costs can vary vastly in different settings, our case series illustrates that diagnostic and therapeutic costs must be evaluated together, preferably within a detailed micro-costing framework, when analyzing how to efficiently manage uncertain melanocytic lesions. The value of this type of conjunct analysis has already been shown in other diagnostic activities related to melanoma and in other health system contexts [21,37]. 

## 5. Conclusions and Limitations

In our case series, *TERT*p mutation analysis was effective in reducing diagnostic uncertainty in 38% (58/151) of the uncertain lesions. Moreover, in our cohort of 151 patients with an initial diagnosis of uncertainty, it lowered the total cost associated to the management of uncertain melanocytic lesions by 36.158 €. An important contribution and differential feature of our work is that it is based on real clinical practice, and it assessed effectiveness and cost-effectiveness within the same diagnostic workflow. A limitation is that diagnostic and clinical costs might vary greatly in different settings. In addition, the two most frequent mutations in *TERT*p were analyzed (i.e., c.−146 C > T and c.−124 C > T), whereas the less frequent c.138_139delinsTT was not. As such, when the *TERT*p mutation analysis was negative, this was negative for c.−146 C > T and c.−124 C > T, but not necessarily for other mutations, such as c.138_139delinsTT. Expanding the *TERT*p analysis to include a ddPCR assay increases the costs but may enable more precise classification and prognostic evaluation of melanocytic lesions. The costs are based on the analysis of one or two samples per run, but the analysis of >2 samples in same ddPCR run can decrease costs by at least 33%. This demonstrates that increasing the volume through the centralization of molecular testing, preferably in melanoma expert centers, will enable lower costs. Further research is needed to gain a better understanding of the utility and efficiency of (expanded) *TERT*p mutation analyses and more comprehensive panels that cover several biomarkers in different lesion subgroups, as well as to analyze the cost-effectiveness of different molecular techniques. 

## Figures and Tables

**Table 1:** Estimation of the probabilities.

Author and Year	Number of Cases	P (*TERT*p Mutation Status+|Malignant) ^1^	P *TERT*p Mutation Status−|Malignant) ^2^
Griewank et al., 2013 [19]	38	31%	69%
Griewank et al., 2014 [20]	362	43%	57%
Populo et al., 2014 [21]	116	22%	78%
Egberts et al., 2016 [22]	96	49%	51%
Nagore et al., 2016 [23]	300	42%	58%
Ofner et al., 2017 [24]	40	55%	45%
Roh et al., 2017 [25]	88	17%	83%
Zehir et al., 2017 [26]	164	75%	25%
de Unamuno Bustos et al., 2018 [27]	170	31%	69%
Tan et al., 2019 [28]	133	32%	68%
	Total cases	Weighted average	Weighted average
	1507	41.22%	58.77%
		Estimated probability P (*TERT*p mutation status+|Malignant)	Estimated probability P (*TERT*p mutation status−|Malignant)
		0.41	0.59

^1^ Prevalence of mutated (positive) *TERT*p status in melanoma. ^2^ Prevalence of wild-type (negative) *TERT*p status in melanoma.

**Table 2:** Demographics.

Patient Characteristics	
Total cases	367
Sex	
Female	225 (61.3%)
Male	142 (38.7%)
Age range	0–90
Mean age	44.3 (SD = 21.4)
Median age	43 (CI 95% [40.8–45.2])
Mean age in cases with *TERT*p-MA performed	40.2
Median age in cases with *TERT*p-MA performed	38
Location	
Extremities	153 (41.7%)
Trunk	120 (32.7%)
Head and neck	52 (14.2%)
Acral	42 (11.4%)

Notes: *TERT*p-MA = *TERT* promoter mutation analysis.

**Table 3:** Lesions grouped according to preliminary histopathological diagnosis.

Group	*n*	Lesion Types
Benign	115 (31.3%)	Compound, junctional, or dermal nevus nevocellularis Acral nevus Halo nevus BAP-1 inactivated nevus Cellular blue nevus Spitz nevus Deep penetrating nevus
Malignant	77 (21%)	Superficial spreading melanoma Nodular melanoma Acral melanoma Lentigo maligna melanoma Desmoplastic melanoma
Uncertain	175 (47.7%)	
Likely benign	49 (28%)	Dysplastic nevus Acral nevus with dysplasia Atypical spitz tumor
Likely malignant	39 (22.3%)	Preferentially a melanoma versus an intermediate lesion of uncertain malignant potential
Ambiguous	87 (49.7%)	IAMPUS (intraepidermal atypical melanocytic proliferation of uncertain significance). SAMPUS (superficial atypical melanocytic proliferation of uncertain significance). MELTUMP (melanocytic tumor of uncertain malignant potential).
Total	367	

**Table 4:** Results of the *TERT*p mutation analysis in likely benign, ambiguous, and likely malignant lesions.

	Preliminary Histopathological Diagnosis			Total
*TERT*p mutation status	Likely benign	Ambiguous	Likely malignant	
Mutated	0	14 (6 C228T and 8 C250T)	25 (7 C228T and 18 C250T)	39 (13 C228T and 26 C250T)
Not mutated	44	55	13	112
Total	44	69	38	151

**Table 5:** Predictive probabilities of malignancy given *TERT*p mutation status (P (malignant|*TERT*p +) and P (malignant|*TERT*p −)).

	P (Malignant)		
*TERT*p mutation status	0.25	0.5	0.75
Mutated	0.91	0.97	0.99
Not mutated	0.17	0.37	0.64

**Table 6:** Modifying effect of the *TERT*p mutation analysis using plasma-derived cfDNA on the preliminary diagnosis using a Bayesian approach.

	*TERT*p Mutation Analysis		
Preliminary Histopathological Diagnosis	No Result *	*TERT*p Mutation Status	
		Not Mutated	C228T or C250T
Likely benign (*n* = 49)	5	44	0
P (malignant)		0.17 Likely benign	
Ambiguous (*n* = 87)	18	55	14
P (malignant)		0.37 Ambiguous	0.97 Malignant
Likely malignant (*n* = 39)	1	13	25
P (malignant)		0.64 Likely malignant	0.99 Malignant

* Note: in these cases, *TERT*p mutation analysis was not reliable due to tissue limitations (i.e., an insufficient sample).

**Table 7:** Classification of lesions before and after performing the *TERT*p mutation analysis.

Before *TERT*p Mutation Analysis			After *TERT*p Mutation Analysis
Preliminary Likely Benign	44	44	Likely Benign
Preliminary Ambiguous	69	55 (−14)	Ambiguous
Preliminary Likely Malignant	38	52 (+14)	Likely Malignant

**Table 8:** Comparison of costs between Scenario A (before performing the *TERT*p mutation analysis) and Scenario B (after performing the *TERT*p mutation analysis).

	Scenario A: Before *TERT*p Mutation Analysis	Scenario B: After *TERT*p Mutation Analysis	Modifying Effect After *TERT*p Mutation Analysis
	Implicated costs: COST 1 (5 mm margin re-excision), cost per patient = 1.274 €	Implicated costs: COST 2 (*TERT*p mutation analysis), cost per patient = 570.5 €	
Benign cases	115	159	+44
Uncertain cases	151	0	−96
Likely benign	44	0	−44
Likely malignant	38	0	−38
Ambiguous	69	55	−14
Malignant cases	77	129	+52
Total sum COST 1 (5 mm margin re-excision)	192.345 €	70.070 €	−122.275 €
Total sum COST 2 (*TERT*p mutation analysis)	0 €	86.145 €	+86.145 €
Total Cost	192.345 €	156.215 €	−36.129 €
Cost Saved			36.129 €

## Data Availability

The data presented in this study are available on request from the corresponding author due to privacy reasons.
